# Targeting Immune Cell Checkpoints during Sepsis

**DOI:** 10.3390/ijms18112413

**Published:** 2017-11-14

**Authors:** Naeem K. Patil, Yin Guo, Liming Luan, Edward R. Sherwood

**Affiliations:** 1 Department of Anesthesiology, Vanderbilt University Medical Center, Nashville, TN 37232, USA; yin.guo.1@vanderbilt.edu (Y.G.); liming.luan@vanderbilt.edu (L.L.); edward.r.sherwood@vanderbilt.edu (E.R.S.); 2 Department of Pathology, Microbiology and Immunology, Vanderbilt University Medical Center, Nashville, TN 37232, USA

**Keywords:** sepsis, immunotherapy, T cell exhaustion, PD-1, PD-L1, CTLA-4, BTLA, TIM-3, LAG-3, 2B4, myeloid cells, immunosuppression

## Abstract

Immunosuppression is increasingly being recognized as one of the causes of increased morbidity and mortality during sepsis. Both innate and adaptive immune system dysfunction have been shown to cause an impaired ability to eradicate the primary infection and also lead to frequent occurrence of secondary opportunistic infections. Pre-clinical and clinical studies have shown that inhibitory immune checkpoint molecules, including programmed death-1 (PD-1), programmed death ligand-1 (PD-L1), cytotoxic T lymphocyte antigen-4 (CTLA-4), T cell membrane protein-3 (TIM-3), Lymphocyte activation-gene-3 (LAG-3) and 2B4, are upregulated during the course of sepsis. Engagement of these inhibitory molecules on various immune cells has been consistently shown to inhibit innate immune cell functions (e.g., phagocytosis, cytokine production and pathogen clearance) and also lead to impaired T cell competence. In numerous pre-clinical models of sepsis, therapeutic agents aimed at blocking engagement of inhibitory immune checkpoints on immune cells have been shown to improve innate and adaptive immune cell functions, increase host resistance to infection and significantly improve survival. Therefore, immunotherapy with immune cell checkpoint inhibitors holds significant potential for the future of sepsis therapy and merits further investigation.

## 1. Introduction

Sepsis is one of the most common causes of death among critically ill patients leading to estimated $14 billion in annual health care costs in the United States alone and much more worldwide [1]. Sepsis, defined as life-threatening organ dysfunction caused by dysregulated host responses to infection according to the third international consensus definition for sepsis and septic shock (Sepsis-3), accounts for more than 5.3 million deaths worldwide per annum [2,3]. No definitive therapy that targets the underlying pathobiology of sepsis exists. Thus, antibiotics, fluid resuscitation and organ support remain the mainstay of treatment. Recent clinical studies indicate that septic patients have a high one year mortality rate after hospital discharge, often due to the development of secondary infections [2,4]. Some investigators postulate that late infections in sepsis survivors stems from prolonged immunosuppression [5].

Classically, the time course of sepsis is characterized by pro-inflammatory and anti-inflammatory phases that occur during variable time points after sepsis. However, the typical biphasic initial pro-inflammatory phase followed by anti-inflammatory phase has been refuted by recent reports [6,7]. Some investigators propose that the pro-inflammatory and anti-inflammatory phases co-exist and that the prolonged presence of each phase defines a syndrome of chronic critical illness [8]. One of the hallmarks of sepsis is decreased ability to eradicate primary infection and increased susceptibility to secondary nosocomial infections often caused by opportunistic pathogens [9,10]. Sepsis often leads to significant multi-organ injury causing increased morbidity and mortality, as shown in numerous pre-clinical studies [11,12,13,14]. Immunosuppression is increasingly being recognized as a significant contributing factor for sepsis-induced morbidity and mortality, especially after hospital discharge [4,15,16]. This further highlights the pathological role of immunosuppression during sepsis, as a majority of deaths occur at delayed time points after the initial stabilization of septic patients with aggressive supportive therapy.

**Figure 1:**
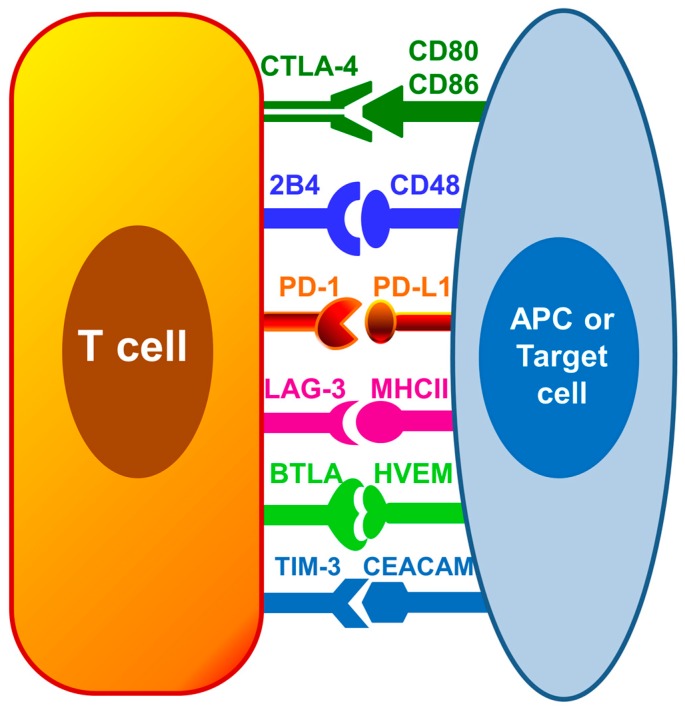
Inhibitory immune checkpoints on immune cells. Interaction among immune cell checkpoint receptors on T cells and antigen presenting cells (APCs) or target cells such as peripheral tissue epithelial cells inhibit leukocyte functions and may contribute to immune dysfunction. PD-1 = Programmed death-1; PD-L1 = Programmed death ligand-1; CTLA-4 = Cytotoxic T lymphocyte antigen-4; BTLA = B and T lymphocyte attenuator; HVEM = Herpes virus entry mediator; TIM-3 = T cell membrane protein-3; LAG-3 = Lymphocyte activation-gene-3; CEACAM = carcinoembryonic antigen-related cell adhesion molecule; MHC II = Major histocompatibility complex II.

Therapies targeting immunosuppression are being intensively researched as a new approach for sepsis treatment. Immune checkpoint receptors including programmed death-1 (PD-1), programmed death ligand-1 (PD-L1), cytotoxic T lymphocyte antigen-4 (CTLA-4) and B and T lymphocyte attenuator (BTLA) have been shown to be increased on immune cells during sepsis and hypothesized to be one of the major contributors causing sepsis induced immune cell dysfunction [17]. These inhibitory immune regulators hinder the immune responses needed to clear off invading pathogens. The focus of this review is to discuss the current knowledge and recent advances regarding immune checkpoints during sepsis, and the future potential for novel immune checkpoints inhibitors, such as anti-PD1, anti-PD-L1 and anti-CTLA-4, as immunotherapeutic agents for restoring host immune response during sepsis. Figure 1 depicts the interaction among known inhibitor immune cell checkpoints on T cells and antigen presenting cells.

### 1.1. Sepsis-Induced Immunosuppression

New treatment protocols with aggressive supportive therapy rescue the majority of the septic patients during the early inflammatory phase, but survivors are then prone to develop an immunosuppressive phase [18]. Opportunistic pathogens such as Pseudomonas, Candida, Acinetobacter and Enterococcus are common culprits for secondary infections among septic patients [10]. Moreover, a high incidence of reactivation of latent viruses such as cytomegalovirus and herpes simplex virus has also been observed [19,20]. Immune cell dysfunction during sepsis not only causes a decreased capability to eradicate primary infection, but also increases the risk for such secondary infections. One of the major mechanisms for immunosuppression is hypothesized to be increased expression of immune regulatory checkpoints including PD-1, PD-L1, CTLA4 and BTLA, and targeting these negative regulators of immune responses has shown to improve host resistance to infections [15,17,21,22]. Although, immunosuppression during sepsis affects both innate and adaptive immune systems, T cell function is known to be the most significantly compromised during sepsis, as a result of the interaction between immune checkpoint receptors such as PD-1/PD-L1 [6,23,24]. The following sections will discuss the individual immune checkpoints and their known roles in sepsis immunopathology.

### 1.2. Programmed Death-1 (PD-1) and Its Ligands PD-L1/PD-L2

PD-1/PD-L1 axis is the most well characterized inhibitory immune checkpoint interaction that has been studied in sepsis immunopathology.

#### 1.2.1. PD-1

Discovered in 1992, the programmed death-1 (PD-1) receptor (CD279) is a 228 amino acid, 50–55 kDa monomeric type I transmembrane glycoprotein belonging to the immunoglobulin superfamily, composed of an extracellular immunoglobulin Variable-type (V-type) extracellular domain, a transmembrane domain, and a cytoplasmic tail which executes the intracellular signaling [25,26]. The intracellular region of PD-1 receptor is composed of ITIM (immuno-receptor tyrosine-based inhibitory motif) and ITSM (immuno-receptor tyrosine-based switch motif) [27]. PD-1 protein is encoded by the *Pdcd1* gene on chromosome 1 in mice and chromosome 2 in humans. Human and murine PD-1 proteins share approximately 60% amino acid identity [28]. T and B cells are the major leukocytes expressing PD-1 receptor, although it is also expressed on monocytes, natural killer cells, and dendritic cells [29]. Programmed death ligand, PD-L1 and PD-L2 are the known ligands for PD-1 receptor.

#### 1.2.2. PD-L1 and PD-L2

PD-L1 (CD274) is also known as B7 homologue 1 or B7-H1, and PD-L2 is also known as B7-DC. PD-L1 is a 33 kDa transmembrane protein, first identified by Dong et al. in 1999 [30], and PD-L2 (CD273) is a 30 kDa transmembrane protein, first identified by Latchman et al. in 2001 [31]. Human and murine PD-L1 and PD-L2 share 69% and 70% amino acid identity, respectively [31]. PD-L1 is known to be expressed both on immune as well as non-immune cells. PD-L1 is not only constitutively expressed but also upregulated upon stimulation on dendritic cells, macrophages, T and B lymphocytes [29,32]. PD-L1 is also expressed in peripheral organs including heart, placenta, lung, liver, pancreas, kidney and tumor cells [29,32]. PD-L2 is more restricted in its distribution, found to be constitutively expressed in dendritic cells and monocytes, and transcripts have also been found in lung, placenta and liver [32].

### 1.3. Role of PD-1 and Its Ligands PD-L1 and PD-L2 in Immune Cell Dysfunction during Sepsis

**Figure 2:**
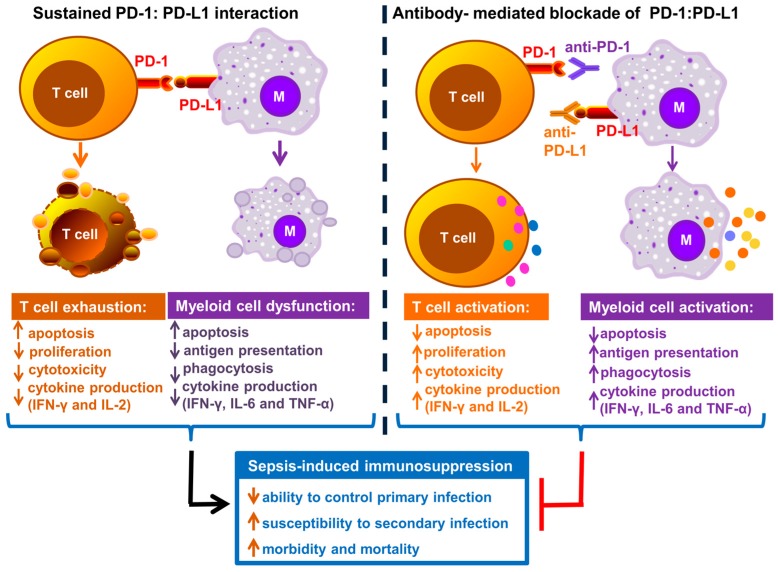
Graphical representation of PD-1–PD-L1 interaction leading to immune cell dysfunction and immunosuppression. PD-1–PD-L1 interaction leads to impaired T cell function (exhaustion) and antigen presenting cell (myeloid) dysfunction. Antibodies targeting each of these inhibitory molecules reverse sepsis induced immunosuppression and improve host resistance to infection. (M = antigen presenting or myeloid cell; PD-1 = Programmed cell death-1; PD-L1 = Programmed cell death ligand-1; IFN-γ = interferon-gamma; IL-2 = interleukin-2; IL-6 = inerleukin-6, upward arrows indicates an increase and downward arrows indicates a decrease).

It is well established that interaction of PD-1 with its ligands causes impaired T cell function. PD-1/PD-L1 induced T cell inhibition represents one of the major inhibitory receptor–ligand interactions studied during sepsis (Figure 2). PD-1 is known to be normally upregulated on the surface of activated CD4^+^ and CD8^+^ T cells to limit their hyper-activation and uncontrolled inflammation [33]. However, sustained up-regulation of PD-1 in the face of high antigen load as a result of severe infection, leads to impairment of both innate and adaptive immune responses [23,34]. The inhibitory immune checkpoint interaction often leads to a phenomenon known as T cell exhaustion. T cell exhaustion may lead to T cell dysfunction causing reduced effector T cell functions, decreased cytokine production, decreased proliferative capacity and apoptosis [34].

Over the past decade, numerous studies have shown a sustained increase in PD-1 and PD-L1 expression on various immune cells during sepsis. Figure 2 depicts the overview of immune cell dysfunction as a result of sustained PD-1–PD-L1 interaction during sepsis and inhibition of this interaction reverses sepsis induced immunosuppression and improves host resistance to infection.

In various pre-clinical studies employing different rodent models of sepsis such as cecal ligation and puncture (CLP) and burn wound infection with *Pseudomonas aeruginosa*, PD-1 expression has been shown to be upregulated on T cells and PD-L1 expression was increased on innate immune cells including monocytes, dendritic cells, Kuppfer cells and neutrophils [17,21,35,36,37,38,39,40,41]. The majority of these studies show that PD-1/PD-L1 axis stimulation during sepsis leads to T cell dysfunction and apoptosis, which is accompanied by increased pathogen burden, multi-organ injury, and mortality. PD-1 knockout has also been shown to improve survival in a neonatal model of cecal slurry-induced sepsis [42]. These findings are further strengthened by clinical studies which also reveal the roles of PD-1 and PD-L1 in immune cell dysfunction during sepsis. PD-1 expression on circulating T cells has been shown to significantly correlate with decreased T cell proliferation and increased secondary infections leading to higher mortality among septic shock patients [43]. Increased PD-L1 expression has also been correlated with increased T cell apoptosis, lymphopenia, and T cell dysfunction [44,45]. A recent notable study by Patera et al. showed that PD-L1 expression was significantly increased on suppressor phenotype subsets of neutrophils and monocytes, and increased PD-1 expression was observed on CD8^+^ T cells among septic patients [24]. Those alterations positively correlated with decreased phagocytic capacity of both neutrophils and monocytes, and decreased CD8^+^ T cell and natural killer (NK) cell function [24]. Wang et al. demonstrated that CLP-induced sepsis caused a significant increase in PD-1 expression on Kuppfer cells in the liver (a type of resident macrophages) and PD-1-deficient Kuppfer cells displayed increased phagocytic capacity and restoration of immune functions [40]. Huang et al. demonstrated a higher percentage of circulating neutrophils positive for PD-L1 expression, which correlated with lethal outcomes [38]. In a postmortem study involving sepsis patients, T cell function was shown to be evidently impaired in association with increased expression of PD-1 receptor and activation marker CD69, and a significant decrease in IL-7 receptor and CD28 expression (a co-stimulatory T cell receptor); and increased PD-L1 and PD-L2 expression on dendritic cells [23]. Taken together, these studies denote that PD-1/PD-L1 axis plays a critical role not only in T cell dysfunction, but also in innate immune cell impairment during sepsis.

Along with bacterial infections, bloodstream fungal infections with Candida has also been shown to cause an increased PD-1 expression on circulating CD4^+^ and CD8^+^ T cells and a decrease in co-stimulatory CD28 expression [46]. Other prospective clinical studies have shown that increased monocyte PD-L1 expression on days 3–4 after sepsis serves as an independent predictor of 28-day mortality in septic shock patients, and septic patients also demonstrated increased expression of PD-1 on CD4^+^ and CD8^+^ T lymphocytes [44,47]. As discussed earlier, PD-L2 expression is restricted to a handful of immune cells, including dendritic cells and monocytes, and there are limited studies addressing its direct role during sepsis-related immunopathology, as compared to PD-L1 which has been extensively studied during sepsis. Table 1 and Table 2 lists the summary of all the pre-clinical and clinical studies which implicate the inhibitory roles of PD-1, PD-L1/PD-L2 and other immune checkpoints during sepsis.

### 1.4. Does PD-L1 Play a Role in Organ Injury during Sepsis?

In addition to immunocytes, PD-L1 (but not PD-1) is also expressed in peripheral tissues including lung, liver, tissue endothelial cells and kidney [29,32]. Therefore, it is important to study the role of PD-L1, if any, with respect to organ injury during sepsis. There are limited studies which demonstrate this aspect of PD-L1. Wu et al. demonstrated that PD-L1 protein expression was significantly increased on intestinal colonic tissue among septic patients as well as in the intestinal tissue of mice subjected to CLP [53]. Furthermore, the preceding study also showed that increased PD-L1 expression on intestinal epithelial cells correlated with intestinal inflammation and increased permeability, signifying dysfunction of intestinal barrier function; and genetic deficiency of PD-L1 or blocking treatment with anti-PD-L1 antibody restored intestinal barrier function. Another study by the same group also showed that genetic deficiency of PD-L1 greatly reduces morphological intestinal injury and mortality induced by CLP in mice [38]. Therefore, it is reasonable to hypothesize that the inflammatory response in the local intestinal milieu is regulated, in part, by PD-1/PD-L1 axis, with PD-L1 on intestinal cells interacting with PD-1 expressed on leukocytes or non-immune cells that express PD-1, or a hitherto unknown binding partner for PD-L1 [38,53].

A study by Zhu et al. showed that CLP induced sepsis caused a significant increase in mRNA and protein levels of PD-L1 in the liver, which was associated with visible morphological damage; and treatment with anti-PD-L1 attenuated the sepsis induced liver injury [50]. From this study, it is not exactly clear if the increased PD-L1 expression was on hepatocytes or the liver resident Kupffer cells or infiltrating immune cells such as lymphocytes, monocytes and other cells. However, a study by Hutchins et al. utilizing a CLP model of sepsis, showed that sepsis caused an increase in expression of PD-L1 on liver sinusoidal endothelial cells, increased liver tissue vascular permeability and edema, and genetic deficiency of PD-L1 restored vascular barrier integrity and attenuated endothelial cell apoptosis [52]. Importantly, the preceding study also demonstrated an increased expression of PD-1 on F4/80^+^ Kupffer cells, which when depleted using clodronate liposomes, also significantly decreased the expression level of PD-L1 on liver sinusoidal endothelial cells. Therefore, it can be hypothesized that during sepsis, increased PD-1 on Kupffer cells might be interacting with increased PD-L1 on endothelial cells leading to detrimental effects on liver vascular permeability and potentially culminating in liver injury and failure. In a study by Zhang et al. evaluating the effectiveness of anti-PD-L1 antibody during CLP induced sepsis, it was noted that treatment with anti-PD-L1 decreased apoptosis of bronchial epithelial cells and alveolar epithelial cells in lungs, although the data was not shown [36]. Using postmortem lung tissue samples, Boomer et al. also showed that PD-L1 expression is detectable on lung parenchymal cells following sepsis [23].

Therefore, these studies demonstrate that PD-L1 plays a role in intestinal and liver injury during sepsis. Further studies need to be undertaken to uncover the exact role of PD-L1 in various organ injuries such as kidney, brain, lung, heart and others during sepsis. If PD-L1 indeed is detrimental for multi-organ function during sepsis, targeting PD-L1 would definitely open up a novel class of therapeutics for sepsis treatment.

### 1.5. Targeting PD-1 and PD-L1 during Sepsis

As discussed above, PD-1–PD-L1 interaction plays a significant role during sepsis-induced immunosuppression. Therefore, targeting either PD-1 or PD-L1 seems a logical approach to restore physiological immune responses and improve outcomes. Antibodies targeting PD-1 and PD-L1, which serve to block the PD-1–PD-L1 interaction, are being extensively studied in pre-clinical models to evaluate their therapeutic efficacy during sepsis. The advent of PD-1/PD-L1 pathway blocking antibodies has been a boon to the field of cancer therapy and numerous PD-1/PD-L1 blocking antibodies from various pharmaceutical companies have been approved by Food Drug Administration to treat human cancers [62]. These therapies have induced successful regression of advanced stage cancers and improved survival rate. Indeed, numerous pre-clinical studies have also shown that targeting PD-1 and PD-L1 during sepsis improves host resistance to infection, which merits further investigation. Table 3, Table 4 and Table 5 summarize the pre-clinical and clinical studies which have evaluated the therapeutic benefit of targeting numerous immune checkpoints.

#### 1.5.1. Targeting PD-L1 during Sepsis

In a study by Zhang et al. treatment with anti-PD-L1 partially attenuated T cell apoptosis and depletion, decreased systemic inflammation, enhanced bacterial clearance, and improved survival [36]. A recent study by Shindo et al. demonstrated that a unique anti-PD-L1 peptide (termed as compound **8**) doubled the survival rate in a two hit model of CLP-induced sepsis followed by Candida albicans-induced fungal infection [55]. Anti-PD-L1 has also been shown to attenuate liver injury in a murine model of CLP induced sepsis [50]. Anti-PD-L1 is effective not only in rodent models of bacterial sepsis but also in rodent models of fungal sepsis. In support of this, a study by Chang et al. showed that even a delayed treatment with anti-PD-L1 up to 24–48 h after the onset of fungal sepsis, reversed T cell dysfunction, increased MHC II on antigen presenting cells and significantly improved survival [51]. A more recent notable clinical study by Patera et al. demonstrated that ex vivo incubation of septic patient’s whole blood with anti-PD-L1 antibody significantly improved phagocytic function of neutrophils and monocytes, and restored CD8^+^ T cell and NK cell functions; with most beneficial effects seen among patient groups with lowest baseline function of these cells [24]. Another study by Chang et al. showed similar findings in that in ex vivo studies, anti-PD-L1 attenuated apoptosis and improved interferon-γ (IFN-γ) and intereukin-2 production by CD8^+^ T lymphocytes from septic patients [45]. A multicenter trial for evaluating the dose safety of anti-PD-L1 (BMS-936559 of Bristol–Myers Squibb) in patients with sepsis has recently been completed and results of the study are awaited (ClinicalTrial.gov# NCT02576457).

#### 1.5.2. Targeting PD-1 during Sepsis

Along with anti-PD-L1, anti-PD1 has also been extensively tested for its therapeutic efficacy in preclinical models of sepsis. In a remarkable study by Brahmamdam et al. anti-PD1 antibody, even when administered 24 h after the onset of CLP induced sepsis, blocked apoptosis and depletion of T lymphocytes and dendritic cells; improved host immune responses; preserved delayed type hypersensitivity responses; and significantly improved survival [35]. Similar to anti-PD-L1, anti-PD1 is also protective during fungal sepsis. Studies by Shindo et al. in a two hit model of CLP followed by Candida albicans fungal sepsis showed that treatment with anti-PD1 improved MHC II expression on splenic macrophages and dendritic cells, and a combination therapy with interleukin-7 increased interferon-γ (IFN-γ) secretion by CD4^+^ and CD8^+^ T cells, and anti-PD-1 had no effect on either proliferation and CD28 expression on CD4^+^ and CD8^+^ T cells [64]. Furthermore, in another study, anti-PD1 was shown to be highly effective in restoring IFN-γ secretion by CD4^+^ and CD8^+^ T cells, MHC II expression on antigen presenting cells and significantly improving survival in a murine primary and two hit model of fungal sepsis [51]. It is noteworthy to mention that the preceding two studies by Shindo et al. and Chang et al. showed benefit with anti-PD1 even when it was administered late after the onset of sepsis, which strengthens its therapeutic implication [51,64]. In addition to animal studies, clinical studies have shown beneficial effects of anti-PD1. Patera et al. showed that anti-PD1 not only improves phagocytic function of innate immune cells like neutrophils and monocytes, but also restored T lymphocyte function, all isolated from the blood of septic patients [24]. Another clinical study by Chang et al. demonstrated that similar to anti-PD-L1, anti-PD1 attenuated apoptosis and improved IFN-γ and intereukin-2 production by CD8^+^ T lymphocytes from septic patients [45].

Therefore, overall the above studies show that blocking the PD-1/PD-L1 interaction with blocking antibodies against each restores immune function among immunosuppressed septic host and provides significant protection.

## 2. Cytotoxic T Lymphocyte Antigen-4 (CTLA-4)

CTLA-4 is also known as CD152, and it is a negative regulator of T cell function, which shares 30% homology to the T cell co-stimulatory molecule CD28 [34]. It is a dimeric cell surface glycoprotein expressed by both activated CD4^+^ and CD8^+^ T cells, and binds to its receptors CD80 (high affinity than CD86) and CD86 (relatively lower affinity than CD80) on antigen presenting cells [65]. CTLA-4 and CD28, both bind to CD80 and CD86, but CTLA-4 binding is of much higher affinity and thereby CTLA-4 counteracts CD28 induced co-stimulation of T cells. The mechanism of action of CTLA-4 to inhibit T lymphocyte proliferation and activation involves a reduction in IL-2 production and IL-2 receptor expression and arresting T cells at the G1 phase of the cell cycle [66,67]. Therefore, conditions which result in sustained upregulation of CTLA-4 compromise T cell immune response and render the host immunosuppressed. Therefore, inhibition of CTLA-4 with blocking antibodies against it might help restore T cell functions in such conditions. CTLA-4 is one of the first immune checkpoints to be clinically targeted in cancer therapy and Ipilimumab (Bristol–Myers Squibb, New York, NY, USA), a monoclonal CTLA-4 antibody, was approved by FDA in 2011 for the treatment of metastatic melanoma [68].

Using a murine model of CLP-induced sepsis, Inoue et al. demonstrated that CTLA-4 expression was progressively increased on both CD4^+^ and CD8^+^ T cells and regulatory T cells, starting at 24 h after induction of sepsis, along with T cell apoptosis and depletion [48]. In the same study, treatment with anti-CTLA-4 inhibited T cell apoptosis by more than 50% and significantly improved survival. Importantly, they also show that protective effect of anti-CTLA-4 is dose dependent and higher doses (200 µg per mouse) worsens the survival outcome as compared to significantly improved survival with lower doses (50 µg per mouse) in two different strain of mice (C57BL6 and CD-1 mice). Anti-CTLA-4 at a low dose (33 µg per mouse) also improves survival in a two hit model of sepsis, comprised of slowly progressive CLP-induced sepsis followed by infection with a fungus, Candida albicans [48]. Therefore, protective effects of anti-CTLA-4 are dose dependent. It is possible that at higher doses, anti-CTLA-4 drives T cell over activation leading to uncontrolled inflammation and deleterious effects of survival. Another study by Chang et al. using two model of sepsis including a primary Candida albicans fungal sepsis and a two hit model (CLP-induced sepsis followed by *Candida albicans*), demonstrated that anti-CTLA-4 increases T lymphocyte IFN-γ production, and significantly improves survival [51]. A prospective clinical study showed that as sepsis progressed, CTLA-4 expression was increased at day 7 on circulating T lymphocytes, as compared to the expression levels at the onset of sepsis [57]. As compared to PD-1–PD-L1 there are limited studies addressing the role of CTLA-4 during sepsis.

## 3. B and T Lymphocyte Attenuator (BTLA)

BTLA is another known inhibitory molecule, which is not only expressed on the surface of T cells but also on innate immune cells including monocytes, macrophages, and dendritic cells [34,69]. BTLA interacts with tumor necrosis factor superfamily molecule termed herpes virus entry mediator (HVEM) and known to cause inhibition of T cell exhaustion [34].

A study by Shubin et al. showed that BTLA expression on T cells correlated with increased mortality in a rodent model of sepsis [49]. In accordance with these findings, Shubin et al. further demonstrated that increased BTLA expression on peripheral CD4^+^ T cells among critically ill sepsis patients positively correlated with development of subsequent nosocomial infections [58]. Furthermore, the preceding study also showed that BTLA deficient mice (*BTLA*^−/−^) had increased numbers of CD4^+^ T cells in the spleen following sepsis and implicated a role for BTLA in apoptosis induced T cell loss. Another study by the same group, using a mouse model and CLP-induced sepsis, also showed that BTLA expression facilitates impairment of innate inflammatory cell activation and promotes MHC II reduction, increases bacterial burden following CLP, increases circulating interleukin-10 levels, and results in multi-organ injury and decreased survival; as compared to septic BTLA knockout mice [49]. Boomer et al. showed that HVEM (ligand for BTLA) is detectable in postmortem lungs of septic patients as compared to controls [23]. On the other hand, a prospective clinical study by Spec et al. showed that the levels of BTLA expression on immune cells was not statistically different among Candida infected septic patients as compared to control patients [46]. Interestingly, anti-BTLA monoclonal antibody is known to be having dual effects including blocking as well as potentiating effects on BTLA mediated effects [70,71]. Only one study has evaluated the effect of ant-BTLA antibody in a two hit model of hemorrhage followed by sepsis [54]. This study by Cheng et al. showed that, treatment with anti-BTLA (at a dose of 25 µg per gram body weight) caused excessive inflammatory immune responses, increased organ injury, leading to significantly increased morbidity and mortality [54]. These results demonstrate that anti-BTLA actually further potentiated BTLA actions in this model of sepsis. In an interesting study by Lange et al. soluble BTLA (sBTLA) levels in the plasma were founds to be significantly higher among sepsis patients as compared to controls and the levels also correlated with clinical severity of the disease [61]. Moreover, the relative risk of 28-day mortality among septic patients was five-fold higher among patients with baseline sBTLA levels of greater than 21 ng/mL, as compared to those with a level below this threshold, suggesting that sBTLA may be explored as a prognostic marker in sepsis [61]. This study has some major drawbacks as noted by the authors including limited plasma samples in certain later phases of the study period, due to missing samples or patients being transferred from the intensive care unit, leading to limitations on conclusions that could be drawn on dynamics of immune markers studied, and varied time points of sepsis onset among different subjects studied. Nonetheless, this is the first study implicating sBTLA as a prognostic marker during sepsis, and further in-depth studies are warranted to further decipher the role of sBTLA during sepsis.

## 4. T Cell Membrane Protein-3 (TIM-3), Lymphocyte Activation-Gene-3 (LAG-3) and 2B4

TIM-3, LAG3 and 2B4 are some of the other known T cell inhibitory molecules which can contribute to T cell exhaustion. TIM-3 interacts with CEACAM1 (carcinoembryonic antigen-related cell adhesion molecule 1) or Galectin 9, and LAG-3 interacts with antigen molecule presented in conjunction with major histocompatibility class II (MHC II) on antigen presenting cells, and 2B4 (also known as CD244) interacts with CD48 on antigen presenting cells [34].

As compared to other inhibitory molecules discussed above, TIM-3, LAG-3 and 2B4 have not been extensively investigated in sepsis yet. Cell surface expression of these inhibitory molecules is not as frequently altered as compared to other classical molecules including PD-1 and PD-L1 [34]. Interaction of TIM3 with its ligand galectin-9 has been shown to cause T cell death and tolerance in vivo [72]. A clinical study by Boomer et al. showed that expression of TIM-3 and LAG-3 was elevated on CD4^+^ T cells among septic patients and LAG-3 was more elevated on CD8^+^ T cells at the onset of acute sepsis phase as compared to TIM-3 [57]. Spec et al. demonstrated that there was no significant difference with respect to TIM-3 expression on CD4^+^ and CD8^+^ T cells during Candida sepsis among critically ill patients as compared to controls, although other inhibitory cell surface receptors including PD-1 and PD-L1 were upregulated and there was a trend towards increase in 2B4 expression on CD8^+^ T cells [46]. However, some recent pre-clinical studies have shown that blocking TIM-3 exacerbates sepsis. A study by Zhao et al. using CLP model of sepsis demonstrated that blocking TIM-3 signaling with soluble TIM-3-Immunoglubulin (sTIM-3-IgG) resulted in exacerbation of sepsis induced macrophage pro-inflammatory responses and lymphocyte apoptosis during acute phase of sepsis, and enhanced anti-inflammatory phenotype for macrophages and CD4^+^ T cells during late phase of sepsis [63]. Furthermore, the preceding study also showed that mice over-expressing TIM-3 attenuated sepsis induced immunosuppression and significantly improved survival, and similar results were obtained upon administering the TIM-3 ligand galectin-9. Previous studies from the same group had also shown that TIM-3 mRNA expression in human peripheral blood mononuclear cells is significantly lower in severe sepsis patients as compared to sepsis patients, and such downregulation of TIM-3 correlated with increased C-reactive protein levels, a clinical marker of patient’s inflammatory status [59]. The same study also showed that blocking TIM-3 signaling using anti-TIM-3 antibody or sTIM-3-IgG increased sepsis severity and significantly decreases survival in a CLP model of sepsis. This finding was correlated with the role of TIM-3 in negatively regulating toll like receptor-4 mediated responses of macrophages leading to inhibition of macrophage activation, and showed that TLR4 signaling pathway is an important mediator of TIM-3 related immune homeostatic mechanisms during sepsis [59]. Based on these studies, it seems that TIM-3 might play a permissive role for protection during sepsis induced immunosuppression, which merits further detailed investigation. However, in all these studies, TIM-3 antibody was administered starting at least one day before sepsis induction and it will be interesting to study the consequences of blocking TIM-3 signaling after sepsis induction. On the contrary, a clinical study by Ren et al. shows that TIM-3 expression on monocytes was significantly elevated among sepsis patients as compared to severe sepsis, septic shock and control patients; and soluble TIM-3 (sTIM-3) levels in the plasma of septic shock group was higher than just sepsis or severe sepsis groups, the levels of which correlated with eventual non-survivors [60]. Therefore, TIM-3 expression on monocytes and sTIM-3 exhibited opposite profiles among patients with varying sepsis severity and detailed mechanistic studies are therefore warranted to delineate the exact role of TIM-3 during sepsis.

In a recent study, Chen et al. demonstrated the inhibitory role of 2B4 during sepsis [56]. This study showed that 2B4 levels are significantly upregulated on T lymphocytes in both animal model of CLP induced sepsis and human sepsis patients as early as 24 h after sepsis induction, along with an increased expression of other T cell exhaustion markers including PD-1 and BTLA. Importantly, administration of anti-2B4 blocking antibody or genetic deficiency of 2B4 significantly improves survival after sepsis. Furthermore, immune cell specific conditional knockout of 2B4 also revealed that specific deletion of 2B4 expressed on CD4^+^ T cell was responsible for enhanced survival as compared to that expressed on CD8^+^ T cells and NK cells. Therefore, 2B4 could be a novel therapeutic target during sepsis and further studies are needed to discover the signaling mechanisms downstream of 2B4 in CD4^+^ T cell during sepsis.

## 5. Blockade of Immune Checkpoints during Sepsis: Is it always Appropriate?

Targeting immunosuppression has opened up a vast field of researching novel therapeutics for sepsis, an area of research which was marred by numerous failed clinical trials targeting pro-inflammatory mediators during sepsis [17,21,73]. However, taking lessons from previous clinical trials during sepsis, it is imperative to understand that no single targeted therapy will fit all the sepsis patients and individualized therapy is the extremely important. Stratifying septic patients based on immune cell phenotyping is one of the ways to determine specific patients who might benefit from immunotherapy. Majority of the clinical research up till now has relied on techniques such as flow cytometry to analyze cell surface expression of inhibitory molecules and ex vivo analysis of immune cells for production of cytokines are predominantly used to determine the state of immune system during sepsis. New technologies such as single-cell mass cytometry might well be the future for high content immune profiling techniques to characterize the phenotype and function of various immune cells during sepsis [74]. Lymphocyte expression of inhibitory receptors including PD-1, BTLA, CTLA-4, TIM-3, LAG-3 and 2B4; downregulation of HLA-DR on monocytes; and upregulation of PD-L1 on innate immune cells such as dendritic cells, monocytes, serve as markers for T cell exhaustion [23] and could very well guide the selection of patients for targeted therapies with individual or a combination of immune checkpoint inhibitors. Appropriate patient selection is the key to finding the right therapy. This was well demonstrated by a recent re-analysis of an original phase III clinical trial data which showed that infusion of recombinant human IL-1 receptor antagonist failed to reduce mortality among severe sepsis patients [75]. However, when the results of the same study were reanalyzed for subgroups of septic patients with characteristics of macrophage activation syndrome, there was a discernible significant positive impact of treatment on survival among this specific group of patients [76]. Another example is the use of anti-TIM-3 antibody during sepsis. Rodent models have shown that treatment with anti-TIM-3 potentiates inflammation and increases mortality during sepsis [63]. Additionally, combination therapies including immune checkpoint inhibitors and other therapies such as monophosphoryl lipid A, interleukin-7, interelukin-15, IFN-γ and FMS like tyrosine kinase-3 ligand might be more appropriate for specific patients and this will lead to better attenuation of sepsis induced immunosuppression [41,77,78,79,80].

### Potential Side Effects of Blocking Immune Checkpoints

We should not forget that an unnecessary inhibition of immune checkpoints can lead to disruption of normal immune homeostasis and could cause inflammatory and auto-immune side effects. Under normal physiological circumstances, immune checkpoints help maintain self-tolerance and inhibit over activation of the T cell response against self-antigens. Clinical trials using immune checkpoint inhibitors have documented immune related adverse effects including fatigue, rash, nausea, pruritus, elevated liver enzymes, abnormal thyroid function, colitis and others [81,82]. Uncontrolled immune activation as a result of immune checkpoint blockade might affect multiple organ systems and result into irreversible lifelong disabilities such as vitiligo, gastro-intestinal disorders, endocrine disorders, polyarthritis, and other autoimmune disorders [82]. Pre-clinical studies in mice have shown that PD-1 deficiency causes an increased incidence of autoimmune pathologies such as lupus like syndromes, de novo type 1 diabetes, dilated cardiomyopathy and others [83,84]. Therefore, caution needs to be exercised when extending anti-PD-1/PD-L1 therapies for sepsis immunotherapy. Therapy that targets immune checkpoints has not been tested yet in septic patients and future research must carefully monitor and address the occurrence of any such adverse effects among septic patients. Once clinical trials are undertaken, subjects should be followed up after hospital discharge to understand not only the short term but also the long term adverse effects, as immune related adverse effects might not fully manifest for months or years. Furthermore, it is important that immune checkpoint blockade therapy should be targeted to patients who actually manifest an increased expression of these receptors. This will require careful monitoring of the phenotype of circulating immune cells among each of the septic patient before starting the therapy. The dose of individual immune checkpoint inhibitors also needs to be well titrated, as a higher dose might precipitate untoward effects and lead to a severe inflammatory response and increased mortality. For example, in murine model of CLP sepsis, anti-CTLA-4 antibody has been shown to be detrimental at higher doses [48]. Well monitored future clinical trials will be able to shed more light on the occurrence and prevalence of untoward effects in sepsis patients.

Hypothetically, targeting immune checkpoints could actually result in severe exaggeration of inflammatory responses if such a therapy is administered during an ongoing severe inflammatory phase of sepsis. Various pathological states including pro-inflammatory, anti-inflammatory and altered responses of immune cells are known to occur simultaneously in septic patients and non-specific boosting of the immune system functions could be detrimental during sepsis [85]. Moreover, murine models of sepsis might not correlate with the exact clinical scenario in septic patients and the majority of the information we have today is based on targeting immune checkpoints in rodent models. For example, the technique of most commonly used murine model of sepsis, CLP, is highly variable among different laboratories depending on the cecum ligation length, amount of fecal matter expressed, fluid/antibiotic resuscitation, and other variable factors. Nonetheless, rodent model do offer a great medium to undertake mechanistic studies during sepsis. A recent study by Patera et al. analyzed the responses to immune checkpoint inhibitors in circulating cells derived from sepsis patients [24], and further study of human patients need to be performed to grow our confidence in using immune checkpoint inhibitors for sepsis therapy before undertaking any larger clinical trials. Table 3, Table 4 and Table 5 summarize the pre-clinical and clinical studies which have evaluated the utility of blocking inhibitory immune cell checkpoints during sepsis.

## 6. Concluding Remarks

There is significant evidence to indicate that immunosuppression plays a detrimental role during sepsis based on numerous pre-clinical and clinical research studies. Targeting immune checkpoints which could potentially reverse innate and adaptive system hypo-responsiveness during sepsis could benefit sepsis patients. However, such a therapy needs to be individualized based on immune status of a particular patient; and cautious treatment with individual or a combination of immune checkpoint inhibitors could be the future of sepsis therapy.

## Figures and Tables

**Table 1:** Summary of pre-clinical studies showing alterations in expression of immune various checkpoints during sepsis.

Reference	Sepsis Model	Alterations in Expression of Immune Checkpoints	Other Major Findings
Huang et al., 2009 [37]	Cecal Ligation and Puncture (CLP)	Increased PD-1 on peritoneal macrophages	**-** Impaired macrophage function (phagocytosis and cytokines) **-** Decreased survival rate
Brahmamdam et al., 2010 [35]	CLP	Increased PD-1 on CD4^+^ and CD8^+^ splenic T cells	**-** Apoptosis of splenic T cells and dendritic cell and decreased survival
Zhang et al., 2010 [36]	CLP	Increased PD-1 on splenic T and B cells and monocytes Increased PD-L1 on splenic B cells and monocytes	**-** Lymphocyte depletion (spleen) **-** Impaired Bacterial clearance **-** Decreased Survival rate
Inoue et al., 2011 [48]	CLP	Increased CTLA-4 on splenic CD4^+^ and CD8^+^ T cells	Increased splenic T cell apoptosis and decreased survival rate
Shubin et al., 2012 [49]	CLP	Increased BTLA and HVEM on macrophages, monocytes, dendritic cells and neutrophils in peritoneum	**-** Decreased peritoneal innate immune cells function (site of infection) **-** Decreased MHC II on macrophages **-** Impaired bacterial clearance
Zhu et al., 2013 [50]	CLP	Increased PD-L1 in liver tissue	**-** Increased levels of liver injury markers **-** Liver injury (histology)
Chang et al., 2013 [51]	**-** Candida fungal sepsis, and **-** Two hit model (CLP + fungal sepsis)	Increased PD-1 on splenic CD4^+^ and CD8^+^ T cells	**-** Splenic T cell dysfunction (decreased IFN-γ) **-** Decreased MHC II on splenic dendritic cells and macrophages **-** Decreased survival rate
Hutchins et al., 2013 [52]	CLP	Increased PD-L1 on liver sinusoidal endothelial cells Increased PD-1 Kupffer cells	**-** Increased liver vascular permeability and injury
Huang et al., 2014 [38]	CLP	Increased PD-L1 on macrophages, monocytes, T and Natural Killer T (NKT) cells and neutrophils	**-** Multi-organ injury, Increased inflammation and decreased survival **-** Inhibition of macrophage function (phagocytosis)
Wang et al., 2016 [40]	CLP	Increased PD-1 on liver Kupffer cells	**-** Decreased MHC II and CD86 expression, and function of Kupffer cells
Patil et al., 2016 [41]	Burn wound sepsis (*Pseudomonas aeruginosa*)	Increased PD-L1 on splenic dendritic cells, macrophages and monocytes No change in PD-1 on splenic T cells	**-** Splenic and circulating lymphocytes depletion **-** Splenic T cell dysfunction (less IFN-γ) **-** Multi-organ injury, impaired bacterial clearance and decreased survival rate
Wu et al., 2016 [53]	CLP	Increased PD-L1 intestinal epithelial cells	**-** Increased intestinal permeability and injury **-** Loss of tight junction proteins in ileum
Cheng et al., 2016 [54]	Two hit model (hemorrhage + CLP)	Increased BTLA on peritoneal macrophages and dendritic cells; and in tissues—ileum, kidney, lung, liver and spleen	**-** Innate immune cell apoptosis (peritoneum) **-** Inflammation, impaired bacterial clearance and decreased survival
Shindo et al., 2017 [55]	Two hit model (CLP + fungal sepsis)	Increased PD-1 on splenic CD4^+^, NKT and NK cells Increased PD-L1 on CD4^+^, NKT and Natural Killer (NK) cells	Significantly decreased survival rate
Chen et al., 2017 [56]	CLP	Increased 2B4 on splenic CD4^+^ and CD8^+^ Increased PD-1 and BTLA on splenic CD4^+^ and CD8^+^	**-** Impaired T cell function **-** T cell apoptosis and depletion **-** Decreased survival rate

**Table 2:** Summary of clinical studies showing alterations in expression of various immune checkpoints during sepsis.

Reference	Sample Size	Alterations in Expression of Immune Checkpoints	Any Other Major Clinical Findings
Guignant et al., 2011 [43]	64 Patients, prospective study	Increased PD-1 and PD-L1 on CD4^+^ T cells, and higher PD-L1/PD-L2 on monocytes	**-** Impaired lymphocyte proliferation **-** Findings correlated with increased nosocomial infections and mortality
Zhang et al., 2011 [44]	19 Patients, prospective study	Increased PD-1 and CD4^+^ and CD8^+^ T cells, and higher PD-L1 on monocytes	**-** Increased T and B lymphocytes apoptosis
Boomer et al., 2011 [23]	Postmortem study, 40 patients	Increased PD-1 on CD4+ and CD8+ on splenic T cells Increased PD-L1 and HVEM on lung tissue Increased PD-L1/PD-L2 on splenic dendritic cells	**-** Depletion of CD4^+^ and CD8^+^ and HLA-DR+ cells in spleen and lung **-** Decreased IL-7 receptor alpha on splenic T cells
Boomer et al., 2012 [57]	24 Patients, prospective study	Increased PD-L1 on splenic dendritic cells, and CTLA-4 on CD4^+^, CD8^+^ T cells Increased TIM-3, LAG-3 on splenic CD4^+^ T cells	Impaired splenic T cell function (as measured by decreased IFN-γ production upon ex vivo stimulation of cells)
Shubin et al., 2013 [58]	24 Patients, prospective study	Increased BTLA on circulating CD4^+^ T cells	Increased BTLA correlated with increased mortality
Yang et al., 2013 [59]	26 Patients (12-sepsis,14-severe sepsis)	Increased TIM-3 mRNA in PBMC’s in sepsis patients as compared to severe sepsis patients	None
Chang et al., 2014 [45]	43 Patients, Prospective study	Increased PD-1 and decreased PD-L1 on CD8^+^ T cells Increased PD-L1 on monocytes	**-** Decreased IFN-γ and Il-12 production by CD8+ T cells upon ex vivo stimulation **-** Increased PD-1 expression on CD8^+^ T cells correlated with increased rate of secondary infections
Ren et al., 2015 [60]	Prospective study; 40-sepsis and42-severe sepsis patients18-septic shock pateints	Increased TIM-3 on monocytes of septic shock patients Decreased plasma soluble TIM-3 levels in septic shock patients	Decreased soluble TIM-3 levels correlated with increased mortality
Patera et al., 2016 [24]	17 Pateints, prospective study	Increased PD-L1 on suppressor neutrophils Increased PD-1 on CD4^+^ T cells and NK cells	**-** Impaired neutrophil, monocyte and NK cell function **-** Impaired CD8^+^ T cell function
Spec et al., 2016 [46]	27 Candida fungal sepsis pateints, prospective study	Increased PD-1 on T cells Trend towards increase in 2B4 on T cells No change in BTLA and TIM-3 expression on T cells	**-** Increased CD69 on CD8^+^ T cells (activated phenotype) **-** Decreased co-stimulatory CD28 expression on CD4^+^ T cells
Shao et al., 2016 [47]	59 Patients, prospective study	Increased PD-L1 on monocytes Increased PD-1 on T cells	**-** Increased PD-L1 on monocytes correlated with severity of sepsis and predictor of 28 day mortality
Wu et al., 2016 [53]	Retrospective analysis	Increased PD-L1 on epithelial cells of colon	None
Lange et al., 2017 [61]	101 Patients, prospective study	Increased plasma soluble BTLA levels (sBTLA)	**-** sBTLA correlated with sepsis severity, and baseline sBTLA >21 ng/mL equated to fivefold higher 28 day mortality rate
Chen et al., 2017 [56]	14 Patients, prospective study	Increased 2B4, PD-1 and CTLA-4 on CD4^+^ T cells	Decreased co-stimulatory ICOS and CD28 on CD4^+^ T cells

**Table 3:** Studies targeting immune checkpoints during sepsis with monoclonal antibodies (pre-clinical studies).

Reference	Sepsis Model	Antibody	Observed Therapeutic Effects
Zhang et al., 2010 [36]	CLP	anti-PD-L1, 200 µg, i.p. route, 24 h before and 2 h after CLP	**-** Decreased apoptosis and restoration of splenic T cell numbers **-** Improved bacterial clearance and survival rate
Brahmamdam et al., 2010 [35]	CLP	anti-PD-1, 200 µg, i.v. route, 24 h after CLP	**-** Decreased splenic T cell and dendritic cell apoptosis and improved function **-** Increased survival rate
Inoue et al., 2011 [48]	CLP	anti-CTLA-4, 50 µg, i.p. route, 6 and 24 h after CLP; and 33 µg, i.p. after fungal sepsis	**-** Decreased splenic T cell apoptosis **-** No effect on ex vivo cytokine production by CD3/CD28 stimulated spleenocytes **-** Improved survival rate
Zhu et al., 2013 [50]	CLP	anti-PD-L1, 50 µg, i.p. route, 1 h after CLP	Attenuation of liver injury (improved histology, and decreased ALT, AST)
Chang et al., 2013 [51]	Candida fungal sepsis, and two hit model (CLP + fungal sepsis)	anti-PD-L1 and anti-PD-L1, 200 µg, i.p. route, 2 days after candida infection anti-CTLA-4, 50 µg, i.p. route, 2 days after candida infection	**-** Increased splenic T cell function (IFN-γ) **-** Increased spleenocyte cytokine production **-** Improved survival
Yang et al., 2013 [59]	CLP	anti-TIM-3, 200 µg, i.p. route, 1 day before and 1, 3, 5 and 7 days after CLP	**-** Increased sepsis severity and systemic inflammation **-** Decreased survival **-** Inhibition of TLR4 mediated macrophage activation and function
Zhao et al., 2014 [63]	CLP	sTIM3-Ig to block TIM-3 signaling, 200 µg, i.p. route, 12 h before, and 48 and 96 h after CLP	**-** Increased macrophage inflammatory response **-** Increased Thymic T cell apoptosis
Shindo et al., 2015 [64]	Two hit model (CLP + fungal sepsis)	anti-PD-1, 200 µg, i.p. route, on day 4 and 8 post CLP	**-** Increased MHC II on splenic dendritic cell and macrophages **-** No effect on splenic T cell proliferation and CD28 expression
Cheng et al., 2016 [54]	Two hit model (hemorrhage + CLP)	anti-BTLA-4, 25 µg/g administered just after CLP	**-** Increased cytokines (KC, MIP-2, MCP-1) in peritoneum **-** Increased peritoneal leucocyte recruitment **-** Organ injury, no effect on bacterial clearance, and significantly decreased survival
Chen et al., 2017 [56]	CLP	anti-2B4, 250 µg, i.p. route, on days—0, 2, 4 and 6, after CLP	**-** Improved splenic T cell function **-** Decreased T cell apoptosis **-** Significantly improved survival
Shindo et al., 2017 [55]	Two hit model (CLP + fungal sepsis)	anti-PD-L1 peptide (compound **8**), 3 mg/kg, s.c route, three times daily from days 5 to 13 after CLP	Significantly improved survival

**Table 4:** Pre-clinical studies employing immune checkpoint knockout/overexpression in mice.

Reference	Sepsis Model	Animal Model	Observed Therapeutic Effects
Huang et al., 2009 [37]	CLP	PD-1 knockout	**-** Improved macrophage function **-** Decreased organ damage and systemic inflammation **-** Augmented bacterial clearance and significantly improved survival
Shubin et al., 2012 [49]	CLP	BTLA knockout	**-** Increased innate immune cell activation **-** Improved bacterial clearance, decreased multi-organ injury and improved survival
Hutchins et al., 2013 [52]	CLP	PD-L1 knockout	**-** Preserved liver vascular integrity **-** Decreased liver sinusoidal endothelial cell apoptosis
Huang et al., 2014 [38]	CLP	PD-1 knockout	**-** Improved macrophage function **-** Decreased inflammation, organ damage **-** Improved survival
Zhao et al., 2014 [63]	CLP	TIM-3 overexpression	**-** Improved macrophage and T cell function **-** Decreased sepsis induced immunosuppression **-** Improved survival
Wang et al., 2016 [40]	CLP	PD-1 knockout	**-** Restoration of MHC II and CD86 on liver Kupffer cells, and increased Kupffer cell phagocytic function **-** Decreased LPS induced apoptosis of liver Kupffer cells
Young et al., 2016 [42]	Neonatal sepsis model using cecal slurry	PD-1 knockout	**-** Increased neutrophil recruitment to site of infection **-** No change in bacterial clearance **-** Increased cytokine response in peritoneum (IL-6, IL-10 and TNF-α) **-** Improved survival rate
Wu et al., 2016 [53]	CLP	PD-L1 knockout	**-** Decreased intestinal (ileum) inflammation and permeability **-** Preservation of tight junction in the ileum

**Table 5:** Evaluating the use of immune checkpoint inhibitors (Clinical studies—ex vivo treatment of isolated immune cells from septic patient’s blood with monoclonal antibodies).

Reference	Patient Population	Antibody Used	Observed Therapeutic Effects
Zhang et al., 2011 [44]	Prospective clinical study with 19 septic patients	anti-PD-L1 antibody	**-** Decreased T cell apoptosis **-** Increased monocyte cytokine production and function
Chang et al., 2014 [45]	Prospective study with 43 septic patients	anti-PD-L1 antibody and anti-PD-1 antibody	**-** Decreased T cell apoptosis **-** Increased T cells IFN-γ and IL-12 production **-** Improved T cell function
Patera et al., 2013 [24]	Prospective study with 17 septic patients	anti-PD-L1 antibody and anti-PD-1 antibody	**-** Restoration of neutrophil, monocyte, T cell and NK cell function **-** Significantly reversing sepsis induced immunosuppression

## Abbreviations

PD-1	Programmed death-1
PD-L1	Programmed death ligand-1
PD-L2	Programmed death ligand-2
CTLA-4	Cytotoxic T lymphocyte antign-4
BTLA	B and T lymphocyte attenuator
HVEM	Herpes virus entry mediator
TIM-3	T cell membrane protein-3
CEACM	Carcinoembryonic antigen-related cell adhesion molecule
IFN-γ	Interferon-gamma
sTIM-3-Ig	Soluble TIM-3 Immunoglobulin
NK cell	Natural Killer Cell
CLP	Cecal ligation and puncture
